# Tribological Properties of Solid Lubricant WS_2_ in Dimples on the Cylinder of Diesel Engine at High Temperature

**DOI:** 10.3390/ma15228161

**Published:** 2022-11-17

**Authors:** Jingguo Fu, Dengqing Ma, Liyang Fan, Zhiwei Yu, Huabing Yin, Chunsheng Ma

**Affiliations:** 1 Shenzhen Academy, Dalian Maritime University, Guangzhou 518063, China; 2 College of Marine Engineering, Dalian Maritime University, Dalian 116026, China; 3 Key Laboratory of Marine Power Engineering & Technology (Ministry of Transport), Wuhan University of Technology, Wuhan 430063, China; 4 COSCO SHIPPING Specialized Carriers Co., Ltd., Guangzhou 510623, China

**Keywords:** solid lubricant WS_2_, textured cylinder, high temperature, releasing performance, tribological properties

## Abstract

Solid lubricant WS_2_ was encapsulated in the dimples on the cylinder surface by the hot-pressing method. The tribological and releasing performance of the as-prepared sample were investigated under high temperature conditions. The results indicate that, compared with the original cylinder, WS_2_ in the dimples exhibited better tribological properties at high temperature than at room temperature. The average friction coefficients of the as-prepared samples were about 0.13 and 0.15 at high temperature and room temperature, respectively, which were 27.8% and 16.7% lower than that of the original cylinder, respectively. Moreover, compared with the original cylinder, the anti-adhesion time of the as-prepared sample increased 2.3-fold. Additionally, the reduced viscosity of the lubricating oil caused by high temperature accelerated the erosion effect and release rate of the solid lubricant in the dimples. Thus, the polar additives in the lubricating oil and the chemical reactions between the cylinder substrates and solid lubricants that WS_2_ released from the dimples are the main factors in friction reduction. This study provides some guidance for anti-friction design of cylinders under high temperature conditions.

## 1. Introduction

Moving parts, such as cylinders and piston rings, tail shafts and bearings, crankshafts and main bearings, etc., are commonly used in the field of ship and ocean engineering. About 70% of the failures in diesel engines are related to the friction between these moving parts [1]. In addition, these parts also consume a lot of energy during operation. Taking the cylinder and piston ring as an example, the friction loss accounts for about 10–20% of the total mechanical energy loss of the entire diesel engine [2]. Therefore, under the global carbon emission control conditions, methods to reduce friction loss and avoid the failure of these moving parts are of great significance to improve equipment energy utilization and save energy consumption.

In order to reduce the friction of these moving parts, researchers have proposed and implemented a variety of methods for friction pairs, such as adding functional additives into lubricating oil [3,4,5], preparing low-friction coatings on the surface of bearing shells [6], manufacturing micro-textures on the bearing surface or cylinder [7,8], and so on. Among these technologies, surface texture has been widely investigated for its easy preparation [9,10] and better anti-friction characteristics, such as storage of lubricating oil to form and maintain the oil film [11], collection of abrasive particles [12], and so on.

However, for the friction pair of cylinders and piston rings, which work at high temperatures, these methods have limitations in reducing friction, especially at the top dead center of the cylinder. The reasons for this are as follows: firstly, the lubricating oil is difficult to transfer to the top dead center during running. Secondly, even if a certain amount of lubricating oil remains on the surface by using the technology of surface texture, the viscosity of lubricating oil decreases due to the high temperature, resulting in difficulty in forming the oil film on the surface. Additionally, the lubricating oil will lose its lubricating effect because of its oxidation and decomposition at high temperature.

Therefore, researchers have replaced the liquid lubricant with solid lubricant, which has higher temperature resistance, by filling them into the micro-texture on the surface [13]. This strategy exhibits a good anti-friction effect, especially under dry sliding conditions. For example, Li et al. [14] found that the combination of WS_2_ films and surface textures on sol-gel ZrO_2_ coatings can improve its anti-friction and anti-wear properties under dry sliding conditions. Dheeraj et al. [15] implemented this strategy on a drilling tool, and found that filling the micro-texture on the drilling margin and flute surface with solid lubricants could improve the machine accuracy of drilling holes in aluminum alloys. Liu et al. [16,17] deposited graphene oxide and fluorinated graphene with MoS_2_ nano-sheets in the surface micro-texture of 304 stainless steel, and tested the tribological properties through a ball-on-disk tester under dry friction conditions. The results show that the synergistic effect between different solid lubricants can greatly improve the friction and wear resistance of the samples. Additionally, our previous study showed that the solid lubricant WS_2_ combined with dimples on the cylinder surface of the diesel engine can enhance the tribological properties at room temperature [18].

Nevertheless, most of the current research is carried out under room temperature and dry sliding conditions. It is well known that the friction between the cylinder and the piston ring of diesel engine occurs under oil lubrication and high temperature conditions. Moreover, the solid lubricant deposited in the micro-texture may be quickly released due to the eroding effect of the lubricating oil. Therefore, in this work, the anti-friction, anti-adhesion, and releasing performance of the solid lubricant in the dimples was investigated under oil lubrication and high temperature conditions by a reciprocating friction and wear tester. The results show that the solid lubricant in the dimples exhibited better anti-friction properties at high temperature than that at room temperature.

## 2. Experimental Details

### 2.1. Preparation of Samples

**Figure 1:**
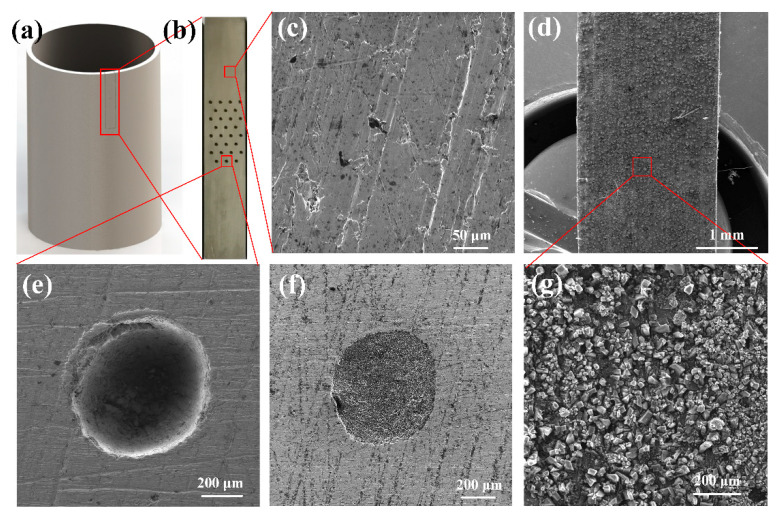
The schematic of the prepared cylinder samples: (**a**,**b**) the schematic of the cylinder cutting process, (**c**) the morphology of the surface of original cylinder, (**d**,**g**) the morphology of surface of the piston ring, (**e**) the morphology of the dimple before filling WS_2_, (**f**) the morphology of dimple after filling WS_2_.

Nodular cast iron cylinder (inner diameter: 110 mm, thickness: 9 mm, roughness: 0.535 μm, hardness: 187 HB) and molybdenum sprayed cast iron piston rings (diameter: 110 mm, thickness: 2.5 mm, roughness: 2.131 μm) of four-stroke diesel engines were selected in this work. The element content of the cylinder is shown in Table 1. The original surfaces of cylinder and piston ring are shown in Figure 1c,g.

First, the complete cylinder was cut into several strip samples by an electro-discharge cutting machine (DK-7732, Taizhou Jiangzhou CNC Machine Tool Co. Ltd., Taizhou, China). Second, a laser etching machine (SHGX-20, Shanhe Precision Equipment Manufacturing Co. Ltd., Shenzhen, China) was used to fabricate dimples on the inner surface of the cylinder, as shown in Figure 1a,b. According to our previous work [12], the parameters of the laser etching machine, including average power, frequency, traverse speed and laser pulse number, were set to 20 W, 20 kHz, 300 mm/s and 30, respectively. The distance between each two adjacent dimple centers was 2 times the dimple diameter. The diameter and depth of the dimple were about 0.8 mm and 430 μm, respectively. The burrs around the dimples caused by laser etching were polished away with 2000# sandpaper, and samples were ultrasonically cleaned in absolute ethanol for 20 min before drying in hot air. Finally, tungsten disulfide (WS_2_) particles (2–5 μm, Aladdin) were deposited in the dimples by the hot pressing method. The redundant WS_2_ on the inner surface was also polished away. The morphologies of dimples before and after filling are shown in Figure 1e,f. The prepared sample was marked as TCW (textured cylinder with WS_2_). The original cylinder sample (marked as OC) and the only textured cylinder sample (marked as TC) were also tested for comparison.

### 2.2. Friction and Wear Test Characterization

**Figure 2:**
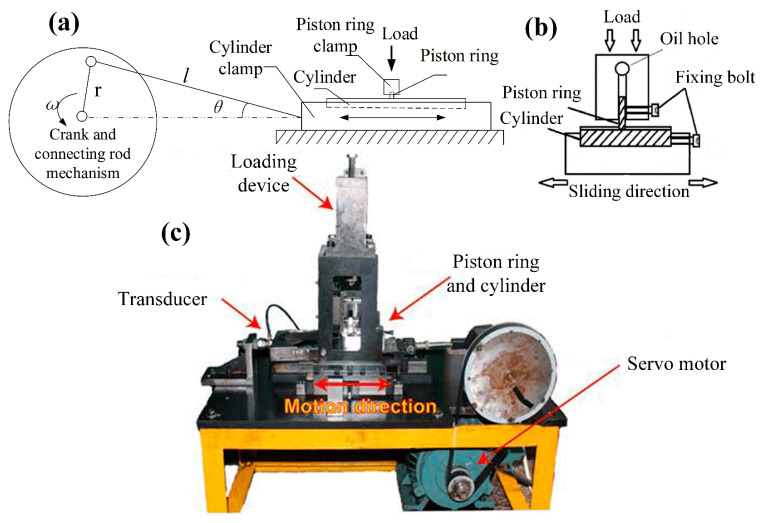
The schematic diagram and picture of the friction and wear tester. (**a**) the schematic of the friction and wear tester; (**b**) the schematic of the assembling jig; (**c**) the picture of the friction and wear tester.

The tribological performances of the cylinder samples were investigated by a homemade reciprocating friction and wear tester at an oscillating amplitude of 30 mm. The piston ring sample was fixed on the upper clamp where the load was applied, and the whole device was fixed on the bedplate by a sliding guide rail. The cylinder strip sample was fixed on the lower clamp with an electric heater unit inside, and its relative sliding movement was converted from the rotational motion of the motor, as shown in Figure 2. A friction transducer was mounted on a structure that connected with the piston clamp and the loading device. The contact area between the cylinder and piston ring can be calculated through the width of the cylinder sample multiplied by the thickness of the piston ring. The applied force can be calculated through the required contact pressure and the contact area. When a relative motion occurred between the cylinder and the piston ring, the instantaneous friction force can be transmitted to the transducer through the structure. The electrical signal from the transducer was amplified by the preamplifier and collected by Labview software (Version 2018) on a computer. The data collection frequency was set to once per second, and only the largest friction force was recorded continuously for the convenience of data analysis.

In order to simulate the real working conditions between the cylinder and piston ring, the temperature of the cylinder strip was heated to 200 °C. The rotating speed of the motor was set to 200 rpm (the maximum sliding velocity was 1.2 m/s). The supply of lubricating oil (Shell SN/GF-5) was 0.25 mL/min. The friction and wear tests were performed under step loading conditions. The initial load of 5 MPa corresponds to 105 N and the working load of 30 MPa corresponds to 630 N. As for the anti-adhesion test, the oil was cut off after 30 min of the running-in period and 30 min of friction and wear test, and the time from the oil cut-off to the sudden increase of the friction force was recorded. The detailed loading and lubrication parameters of the experiments are shown in Table 2. Each test was done at least two times until the friction force was almost the same two measurements. The friction and wear tests at room temperature were also performed for comparison.

### 2.3. Characterization

The morphologies of the prepared cylinder and piston ring samples before and after the friction and wear test were observed by Scanning Electron Microscopy (MIRA3, Tescan, Brno, Czech Republic) and the elemental composition was analyzed by an Energy Dispersive Spectrometer (UltimMax40, Oxford Instruments, Oxford, UK) equipped on the Scanning Electron Microscope. The depth of the dimples on the cylinder surface and the profiles of the piston ring samples were measured by a three-dimensional surface profiler (OLLE5-74S14/7E-2, Olympus, Tokyo, Japan). The chemical composition of the worn surface was analyzed by X-ray Photoelectron Spectroscopy (PHI5000 Versa Probe III, Ulvac-Phi, Japan). The data were calibrated with the saturated C1s peak at 284.8 eV. XPS Peak Fit software (Version 4.1) was used for handling XPS data and fitting the curve of the peaks.

## 3. Results and Discussion

### 3.1. Tribological Properties

**Figure 3:**
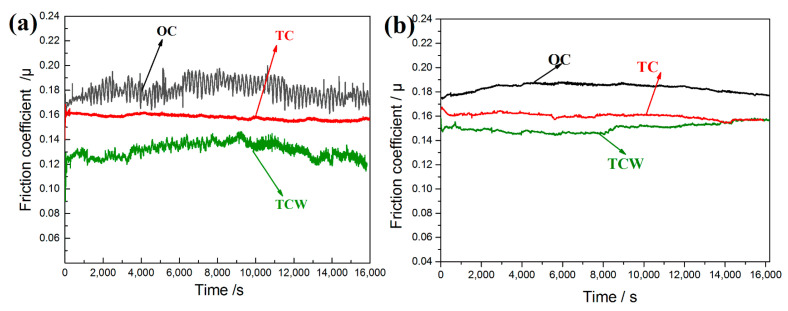
The friction coefficient of different cylinder samples at different temperatures after running-in stage; (**a**) at the temperature of 200 °C, (**b**) at room temperature.

Figure 3 shows the friction coefficient of different cylinder samples at different temperatures during the friction and wear tests. It can be seen that the friction coefficient of the OC sample was the largest, and the friction coefficient of the TCW sample was the smallest, whether at high temperature or normal temperature. The average friction coefficients of the OC samples were almost the same at different temperatures, at about 0.18. The overall variation tendency of the friction coefficients was similar, increasing first and then decreasing. However, it can be clearly seen that the friction coefficient of the OC sample fluctuated greatly at high temperature, while it was relatively stable at room temperature. For the TC samples, the average friction coefficients were also the same, at about 0.16. Furthermore, both the friction coefficients were stable and smooth. The variation tendency was gradually decreasing. As for the TCW samples, the friction coefficients were obviously different at different temperatures. The average value was about 0.15 at room temperature, while it was only about 0.13 at high temperature. Additionally, the friction coefficient also showed a downward trend at high temperature; however, the friction coefficient experienced an upward trend at room temperature. Compared with the OC sample, the average friction coefficient of TCW samples at high temperature decreased by 27.8%, while the value only decreased by 16.7% at room temperature.

From the above, it can be deduced that the textured cylinder filled with solid lubricant can significantly reduce the friction coefficient, especially at high temperature. The reason may be that at high temperature, the viscosity of the lubricating oil becomes lower, and it is difficult to form a continuous lubricating oil film on the friction surface, which makes the friction coefficient of the OC sample fluctuate. When there are dimples on the cylinder surface that can store lubricating oil, the continuity of the lubricating oil film is improved. The fluctuation, as well as the value of the friction coefficient, is also reduced in the TC sample in Figure 3a. However, when the solid lubricant, WS_2_, with low sensitivity to temperature, is stored in the dimples, it can be released from the dimples under the reciprocating scraping action of the piston ring, forming a continuous solid lubricating film, which has an excellent anti-friction effect at high temperature.

**Figure 4:**
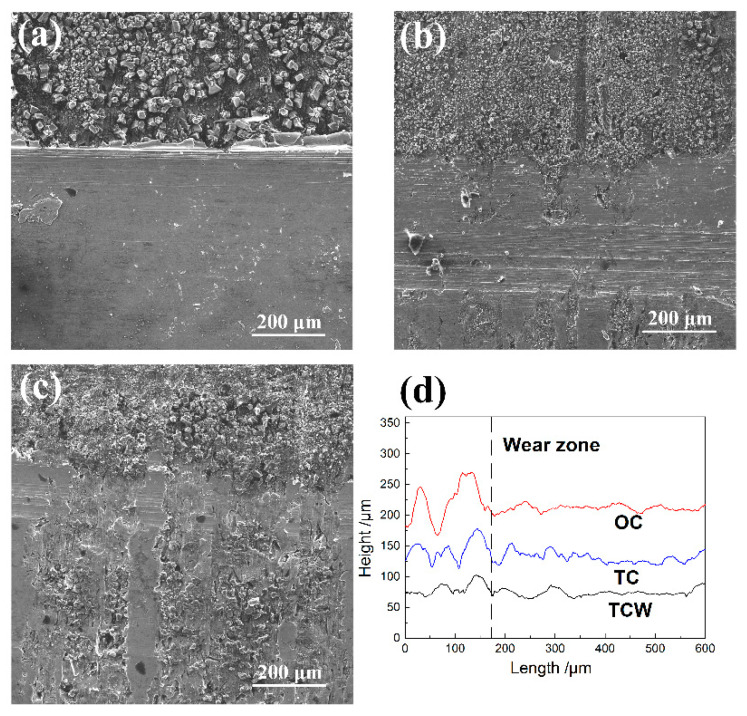
Morphologies and profiles of the piston ring samples; (**a**) the surface morphologies of the piston ring corresponding to the OC sample, (**b**) the surface morphologies of the piston ring corresponding to the TC sample, (**c**) the surface morphologies of the piston ring corresponding to the TCW sample, (**d**) the profiles of the worn surface of the piston ring samples.

Due to the release of solid lubricant from the dimples on the surface during the experiment, the weight difference of the cylinder sample before and after the test includes the amount of wear of the cylinder sample and the amount of solid lubricant released. Therefore the weighing method cannot be adopted to characterize the wear amount of the cylinder sample. In addition, the wear amount of the cylinder is much smaller than that of the piston ring. Therefore, the wear amount of the piston ring sample, namely the height difference between the worn area and the unworn area, was employed to characterize the tribological properties to improve the accuracy of the test, as shown in Figure 4. It can be seen from Figure 4a,d that a micro-convex body was formed on the edge of the worn area of the piston ring corresponding to the OC sample, and the height of the micro-convex body was about 120 μm. Nevertheless, the worn area was relatively flat, indicating that serious wear occurred. The height of the micro-convex body of the piston rings corresponding to the TC and TCW samples were about 90 μm and 50 μm, respectively, as shown in Figure 4b,c. Therefore, the results show that the solid lubricant in the dimples on the surface of the cylinder can extremely improve the tribological properties of the cylinder and piston ring.

### 3.2. Friction Mechanisms

**Figure 5:**
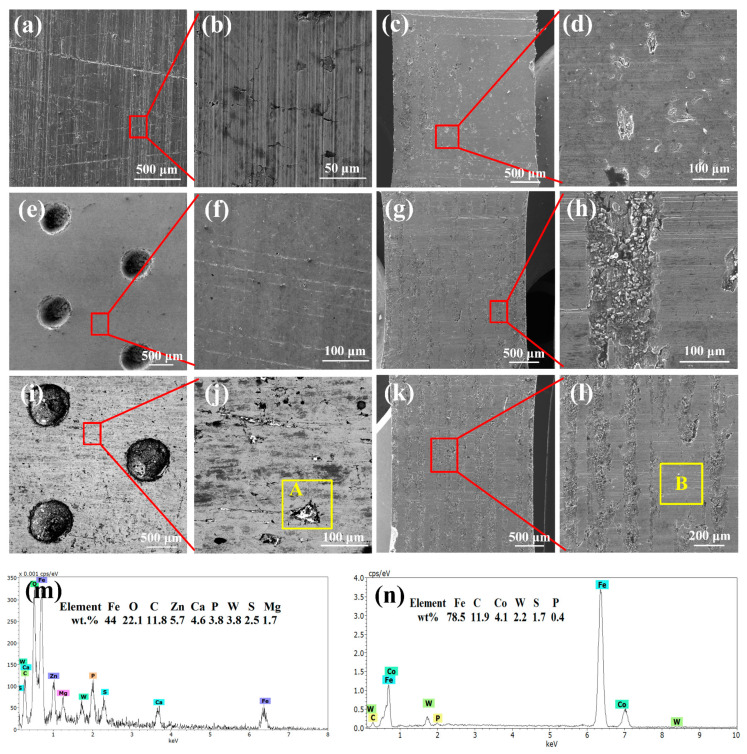
Morphology and EDS result of different samples after testing at high temperature; (**a**,**b**) the surface morphologies of the OC sample, (**c**,**d**) the surface morphologies of the piston ring corresponding to OC sample, (**e**,**f**) the surface morphologies of the TC sample, (**g**,**h**) the surface morphologies of the piston ring corresponding to TC sample, (**i**,**j**) the surface morphologies of the TCW sample, (**k**,**l**) the surface morphologies of the piston ring corresponding to TCW sample, (**m**) the EDS result of area A, (**n**) the EDS result of area B.

Figure 5 shows the surface morphologies of different cylinder and piston ring samples after the friction and wear test at high temperature. It can be seen from Figure 5a,b that after the test, there were few honing patterns on the surface of the OC sample. Additionally, many shallow scratches appeared on the surface, indicating slight adhesive wear occurred on the surface, which is also consistent with the fluctuation of the friction coefficient in Figure 3a. Meanwhile, the coating on the piston ring surface was completely rubbed off, as shown in Figure 5c,d. The reason may be that the low viscosity of lubricating oil caused by the high temperature makes it difficult to form oil film between the cylinder and the piston ring sample. Furthermore, the main types of friction are boundary friction and dry friction. For the TC sample, we note the lack of honing patterns and only a small amount of coatings that still existed on the cylinder surface and piston ring after the test, and that no obvious scratches were seen on the surfaces except for some small abrasive particles on the cylinder surface, as shown in Figure 5e–h. This demonstrates that the lubricating oil stored in the dimples can improve the lubrication state between the cylinder and the piston ring [19].

**Figure 6:**
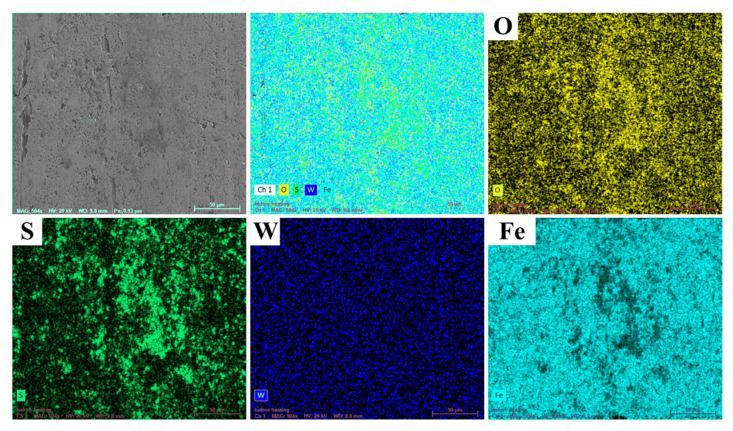
The EDS results of the worn surface of the TCW sample.

However, for the TCW sample, in addition to the honing patterns on the surface, some residual material was also found in the honing patterns, as shown in the region A in Figure 5j. The EDS results of region A (Figure 5m) indicated that it was WS_2_. Moreover, there was also a noticeable amount of solid lubricant WS_2_ remaining inside the dimples. It demonstrates that some of the WS_2_ released from the dimples is stored in the surface honing patterns. As for the corresponding piston ring, much more coating remained on the surface, as shown in Figure 5k,l. The EDS results of region B (Figure 5n) demonstrated that some WS_2_ was also transferred to the piston ring surface. Since the solid lubricant WS_2_ has a layered structure and low intermolecular shear strength, it can form an intermittent solid lubricating film on the friction surface, exhibiting excellent anti-friction performance [20]. This also explains the small and smooth friction coefficient of the TCW sample in Figure 3a. Furthermore, the EDS results of area A also showed that, in addition to the matrix element Fe, there were a higher content of O and a lower content of Zn, P, Mg and Ca elements on the surface. The oxygen may be derived from iron oxides which are formed during the friction process. The Zn, P, Mg and Ca elements indicate that, in addition to the solid lubricant WS_2_, polar additives in the lubricating oil also participate in friction reduction under high temperature conditions [7]. In addition, the EDS elements mapping results of the worn surface of the TCW sample also showed that S and W were homogeneously distributed over the entire worn surface. Moreover, some mapping areas also showed that S and O overlapped, indicating that some sulphure was oxidized during the test, as shown in Figure 6.

**Figure 7:**
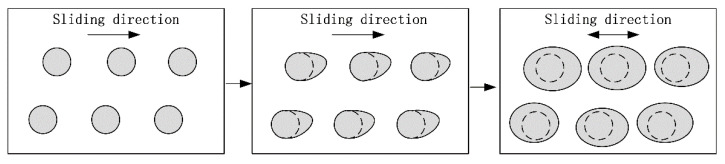
The transfer mechanism of the WS_2_ in the dimples.

Thus, the transfer mechanism can be induced, as shown in Figure 7. Firstly, under the erosion effect of the lubricating oil, the solid lubricant deposited in the dimples will become loose and expansive. Then, the solid lubricant will be scraped onto the surface of the cylinder by the reciprocating piston ring. Under the extrusion action of the piston ring, part of the solid lubricant was stored in the micro-texture on the surface of the cylinder, or directly formed a solid lubricating film on the surface to achieve a continuous anti-friction effect. Meanwhile, part of solid lubricant will be taken away from the friction surface along with the lubricating oil.

**Figure 8:**
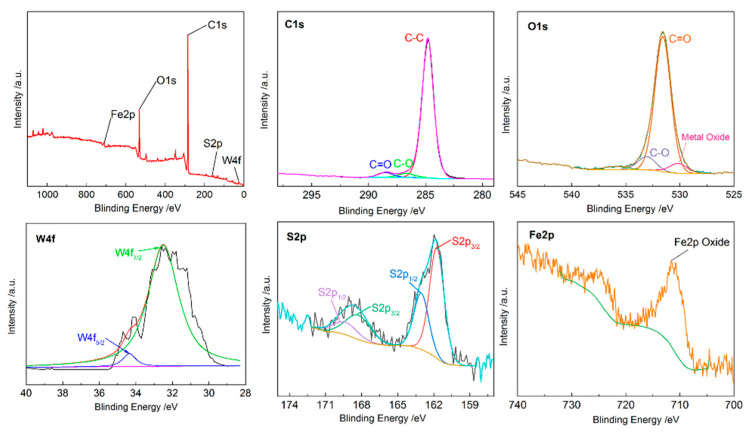
The XPS results of the worn surface of the TCW sample.

In order to further discuss the anti-friction mechanisms, X-ray Photoelectron Spectroscopy (XPS) was performed on the worn surface. The bonding energies of W4f, S2p, Fe2p, O1s, and C1s were measured to elucidate the chemical bonding on the surface. The results are shown in Figure 8. There are two peaks at 32.5 eV and 34.7 eV in W4f, indicating the existence of WS_2_ on the surface [21]. Moreover, the peak of the metal oxides of S2p at 161.6 eV combined with the peak of the oxide of Fe2p at 712.2 eV demonstrate the formation of FeS. The peak in S2p at 168.2 eV proves the presence of SO_2_ [22]. In addition, the peak of oxide of Fe2p at 710.8 eV combined with the peak at 530.2 eV belonging to O1s give evidence for the occurrence of Fe_3_O_4_ during the friction and wear test [23]. However, the peaks at 284.8 eV, 286 eV and 288.5 eV of C1s and the peaks at 531.5 eV and 533 eV of O1s indicate the presence of amorphous carbon and its oxides on the surface, which may be the graphite in the cast iron, or foreign pollutants [24].

According to the above results, the anti-friction mechanisms of WS_2_ encapsulated in the dimples on the cylinder at high temperature are deduced. The solid lubricant WS_2_ is released from the dimples to the friction interface by the scraping action of the reciprocating piston ring. Part of the WS_2_ which has a layered structure forms an intermittent solid lubricating film to achieve an anti-friction effect. Part of the WS_2_ decomposes and reacts with the matrix to form FeS under high temperature and high load conditions, which has some anti-friction and self-healing effects. At the same time, the iron matrix is also oxidized at high temperature.

### 3.3. Releasing Performance of Solid Lubricant

**Figure 9:**
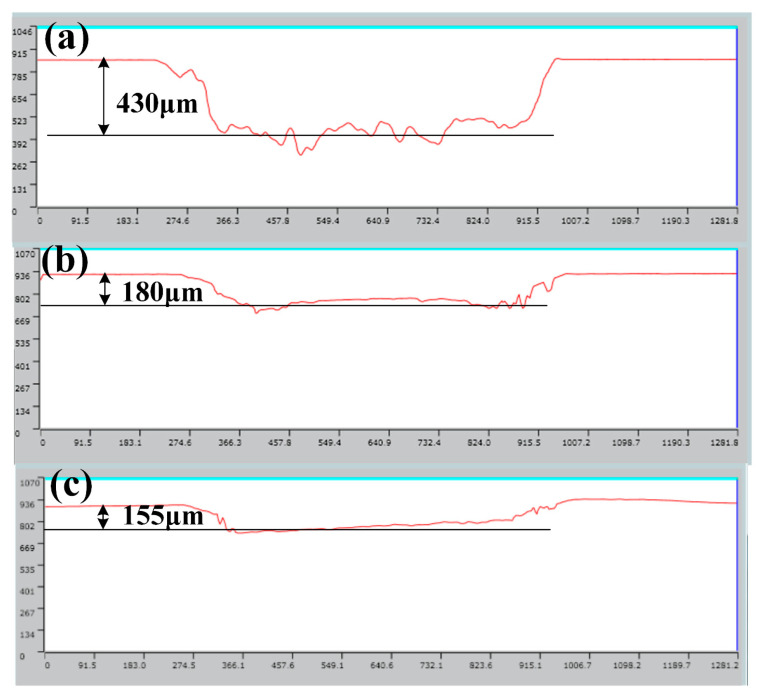
The depth of dimples; (**a**) the original depth of dimple, (**b**) the dimple depth of TCW sample after the test at high temperature, (**c**) the dimple depth of TCW sample after the test at room temperature.

The above friction mechanism showed that the reduction of the friction coefficient at high temperature was mainly due to the release of solid lubricant WS_2_. In order to investigate whether the working temperature affected the release behavior of the solid lubricant WS_2_ in the dimple, a three-dimensional confocal microscope was used to measure the depth of the dimples on the cylinder after the friction and wear test at different temperatures, which indicated the releasing rate of the solid lubricant WS_2_. The results are shown in Figure 9. As can be seen from Figure 9a, the average depth of the dimple after laser etching was about 430 μm. After filling with the solid lubricant, the dimple was covered with solid lubricant, as shown in Figure 1f. However, the solid lubricant WS_2_ in the dimples will be gradually released under the erosion of the lubricating oil and the scraping of the piston ring. The depth of the dimples measured after friction and wear test represented the release amount of the solid lubricant. At high temperature, the depth of the dimple was about 180 μm, which means the release rate was about 36 μm/h, as shown in Figure 9b. At room temperature, the dimple depth and release rate were slightly lower than those at high temperature, at 155 μm and 31 μm/h, respectively, as shown in Figure 9c. The higher release rate of solid lubricant at high temperature may also be one of the reasons for the gradual decrease of friction coefficient under the high temperature conditions, as shown in Figure 3a.

The reason for higher release amount may be that the viscosity of the lubricating oil becomes lower at high temperature, which makes the permeability of the lubricating oil stronger. Thus, it is easier to erode the solid lubricant WS_2_ that was deposited in the dimple, thereby causing it to loosen. Moreover, the solid lubricant WS_2_ expands under high temperature. The loose and expanded solid lubricant WS_2_ is scraped onto the friction surface by the reciprocating piston ring, resulting in the faster release of solid lubricant WS_2_ in the dimple.

### 3.4. Anti-Adhesion Performance

**Figure 10:**
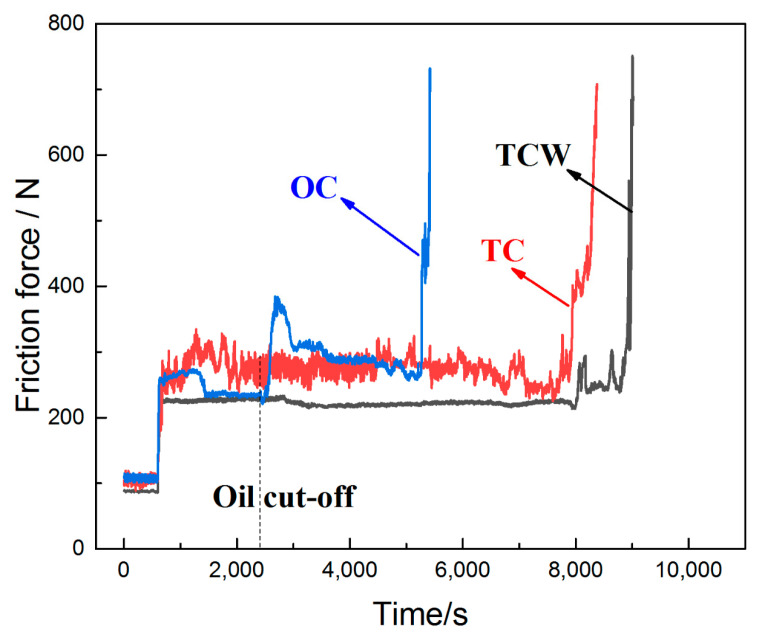
Scuffing time of samples during the anti-adhesion test.

Figure 10 shows the friction force versus scuffing time during the anti-adhesion test. It can be seen that the friction force of the cylinder samples basically remained stable during the running-in period. When the load increased to 630 N, the friction forces of OC and TC samples fluctuated greatly, except for the TCW sample. The fluctuation of the friction forces may mainly be related to the high temperature. Under such conditions, it was difficult to establish a lubricating oil film on the friction surface, and the lubricating state was unstable.

When the oil was cut off, the friction force of the OC sample changed abruptly after about 100 s. Afterwards, it fell back to normal until the adhesive wear occurred. The time from oil cut-off to scuffing was about 2800 s. This phenomenon indicates that the residual oil on the surface can only provide temporary lubrication and the friction state will deteriorate rapidly. The drastic change in the friction force may be related to the polar additives in the lubricating oil which can also be confirmed from the EDS results in Figure 5m. Under high temperature and high load conditions, the polar additives react with the surface to form a chemical reaction film reducing friction force.

Moreover, it can also be clearly seen that the anti-adhesion properties of both the TC and TCW samples were significantly improved. For the TC sample, the friction force fluctuated repeatedly and began to rise sharply 5500 s after the oil cut-off. The repeated fluctuations in friction force are mainly attributed to the lubricating oil stored in the dimples. When the lubricating oil is consumed, adhesive wear will occur. However, for the TCW sample, the friction force remained at a small and stable value after the oil cut-off, and the friction force did not increase drastically until 6500 s after the oil cut-off. Compared with the OC sample, the anti-adhesion time was increased 2.3-fold, showing excellent anti-adhesion performance. This can be attributed to the release of WS_2_, which initially was deposited in the dimples. The encapsulated WS_2_ in the dimples, which has become loose due to the erosion of the lubricating oil, is transferred to the friction surface by the scraping action of the piston ring to form a solid lubricating film. The WS_2_ released from the dimples can reduce friction even after the oil cut-off. Thus, the friction force has been kept at a small and stable value. Adhesive wear occurs until the release of WS_2_ is insufficient to maintain the solid lubricating film on the surface.

## 4. Conclusions

In this work, the anti-friction, releasing performance, and anti-adhesion performance of the solid lubricant WS_2_ deposited in the dimples at high temperature were investigated by a reciprocating friction and wear tester. The results indicate that:

(1) The anti-friction property of the solid lubricant WS_2_ deposited in the dimples at high temperature is better than that at room temperature. The average friction coefficients of the as-prepared samples were about 0.13 and 0.15 at high temperature and room temperature, respectively. Compared with the original cylinder sample, the average friction coefficients of cylinder samples with dimples filled with solid lubricant WS_2_ at high temperature and room temperature decreased by 27.8% and 16.7%, respectively;
(2) The anti-adhesion performance at high temperature is also improved. Compared with the original cylinder sample, the anti-adhesion time of the cylinder samples with dimples filled with solid lubricant WS_2_ increased by 2.3 times;
(3) The reduced viscosity of lubricating oil caused by high temperature accelerated the erosion effect of solid lubricant WS_2_ in the dimples on the cylinder surface, which will enhance the anti-friction effect;
(4) The polar additives in the lubricating oil and chemical reactions between the cylinder substrates and solid lubricants WS_2_ are the main factors for the reduction in friction.

However, the anti-friction effect at high temperature may also have some relationship with the solid lubricants and the parameters of dimples. Therefore, these factors are also worth investigating; some of which will be studied in our following works.

## Figures and Tables

**Table 1:** The main composition of the cylinder.

Element	C	Si	Mn	P	S	Mg	Fe
wt%	3.6	2.3	0.7	<0.1	<0.04	0.03	The rest

**Table 2:** The detailed parameters of the experimental methods at high temperature.

Experimental Methods	Testing Parameters		
	The First Stage (Running-In Period)	The Second Stage	The Third Stage
Friction and wear test	105 N, 30 min, 200 °C, with oil	630 N, 270 min, 200 °C, with oil	-
Anti-adhesion test	105 N, 30 min, 200 °C, with oil	630 N, 30 min, 200 °C, with oil	630 N, 200 °C, oil cut off

## Data Availability

All data that support the findings of this study are available from the corresponding authors upon reasonable request.
